# Embryonic origin of XX/XY chimerism in an in vitro fertilization–conceived individual

**DOI:** 10.1016/j.xfre.2026.01.001

**Published:** 2026-01-17

**Authors:** Anjali Patel, Ecem Esencan, Zameena Lakhani, Eve C. Feinberg

**Affiliations:** a Northwestern Feinberg School of Medicine, Northwestern University, Chicago, Illinois; b Department of Obstetrics and Gynecology, Division of Reproductive Endocrinology and Infertility, Northwestern Feinberg School of Medicine, Northwestern University, Chicago, Illinois; c Department of Obstetrics and Gynecology, Division of Clinical Genetics, Northwestern Feinberg School of Medicine, Northwestern University, Chicago, Illinois

**Keywords:** Chimera, in vitro fertilization, genetic counseling, embryo transfer

## Abstract

**Objective:**

To report an adult case of XX/XY chimera resulting from in vitro fertilization with transfer of three embryos, that was discovered with preconception genetic carrier screening.

**Design:**

Case report.

**Subject:**

A 33-year-old nulliparous woman who was conceived via in vitro fertilization with three embryos transferred presented for embryo banking for future family building.

**Exposure:**

Genetic carrier screening panel.

**Main Outcome Measures:**

Genomic analysis of recessive gene carrier status.

**Results:**

An inconclusive genetic carrier screening result led to further investigation that revealed two distinct cell lines, one containing Y-chromosome material, confirming the diagnosis of chimerism.

**Conclusion:**

There may be an undiagnosed population of human chimeras resulting from the transfer of multiple embryos with assisted reproductive technologies. The biologic and clinical implications of congenital chimerism are incompletely understood.

Chimerism refers to an organism composed of two or more genetically distinct cell lines derived from two or more zygotes (1, 2, 3). The prevalence of naturally occurring chimeras is unknown. Naturally occurring chimeras can be classified into three categories recognized by the literature (1, 3, 4):

(1) Microchimeras contain a minor cell population constituting less than 1% of all cells of the sampled tissue and arise from the transfusion of blood stem cells, such as in feto-maternal cell trafficking across the placenta.
(2) Twin chimeras, also known as blood group chimeras, occur because of cell transfer between dizygotic twin fetuses through blood transfusion via placental vascular anastomoses; stem cells can thereafter migrate to the bone marrow and proliferate into adulthood.
(3) Tetragametic chimeras form by the fusion of two distinct zygotes.

In addition, iatrogenic chimeras can occur in recipients of cells, tissues, and organs, such as those who have received transplants or large volume blood transfusions, after which lymphocytes can survive for decades (3).

Chimerism may be suspected in an individual with two different skin pigmentations following embryonic patterns, binocular heterochromia, or disorders of sex development if the chimera is of mixed sex (XX/XY). The majority of human chimeras appear phenotypically normal and are likely to remain undetected (1, 3, 5, 6, 7). The diagnosis is supported by a detailed clinical history (1, 3, 6, 8, 9, 10). Human twin chimeras are relatively common, with one large study describing a cohort where 8% of twins and 21% of triplets were found to have blood group chimerism (11). However, the phenomenon is observed relatively rarely as detection often relies on incidental findings seen on blood group serologies, parentage testing, or cytogenic analyses that are less frequently completed in healthy individuals (3, 8, 12). In comparison, tetragametic chimerism is a rarely diagnosed entity (3, 4, 5, 6).

Although the increased risk of multiple gestation associated with in vitro fertilization (IVF) is well established (13), discussion regarding the relationship between IVF and human chimerism remains limited to a small number of case reports (6, 7, 14, 15). After the introduction of IVF in the late 1970s, transfer of two or more embryos per cycle was standard practice to enhance pregnancy rates, given the initially low implantation rates (16). As the science of assisted reproductive technologies (ART) advanced, the practice of multiple embryo transfer led to increasing incidence of twin and triplet pregnancies, which rose steadily between 1980 and 1997 (13, 17). In 1998, an effort was made to reduce multiple gestations and the Society for Assisted Reproductive Technology and American Society for Reproductive Medicine (SART/ASRM) Practice Committee published recommendations to limit the number of embryos transferred on the basis of maternal age and treatment prognosis (17, 18, 19). The mean number of embryos transferred in the United States began to decrease, and the rates of triplets and higher-order births decreased, whereas twin births continued to rise (17, 20, 21). Additional guidelines on single embryo transfer in good prognosis patients were published by ASRM and SART to minimize the risk of multiple pregnancy. In 2025, single embryo transfer in good prognosis patients remains the standard of care.

## Case report

A 33-year-old nulliparous woman and her male partner were seen in the reproductive medicine clinic of an academic medical center for fertility preservation consultation, as the couple was interested in embryo banking for deferred reproduction.

The patient reported menarche at age 11 and historically regular menstrual cycles averaging 28 days in length without dysmenorrhea. Levonorgestrel-releasing intrauterine device (Mirena) was inserted for contraception 7 months before consultation and the patient reported irregular bleeding since placement. There was no relevant past medical or surgical history, and she was not using any medications. The patient self-identified as Caucasian. Family history revealed that the patient was conceived via IVF with an anonymous sperm donor. She had both an older brother and a twin brother; both are healthy and neither had yet attempted to have children. The patient did not have any information about the sperm donor’s medical history. There was no other known significant family history.

Her physical examination was unremarkable. Transvaginal ultrasonography showed a normal uterus and bilateral ovaries of normal size and morphology. Baseline laboratory evaluation, including cycle day 3 estradiol, luteinizing hormone, and follicle-stimulating hormone, was normal. Antimüllerian hormone was slightly low for age at 1.32 ng/mL.

The patient underwent venous blood collection for genetic carrier screening using the Horizon 274 panel (Natera, Inc., Austin, TX). The results were reported as inconclusive and repeat testing was performed with the same results. The patient reported that she also received inconclusive results from saliva-based genetic testing using 23 and Me (San Francisco, CA), Inc on two separate attempts in the past. Genetic counseling was recommended. Further detailed history revealed that the patient’s mother underwent multiple IVF cycles, with embryos created using sperm from more than one donor. Transfer of three embryos resulted in a twin pregnancy and the live birth of the patient and her brother. It is unknown whether there was a visible third gestational sac on early ultrasound or whether the patient shared the same sperm donor as her twin sibling.

Further inquiry with Natera, Inc. elucidated that the sample failure occurred because of quality control measures that detected a second cell line in the patient sample. Natera, Inc. confirmed that this contamination occurred before laboratory receipt; common causes noted included recent blood transfusion, bone marrow transplant, or chromosomal abnormalities. The patient did not have a history of either blood transfusion or bone marrow transplant. The raw data from the patient’s blood sample revealed two distinct cell lines, and Y-chromosome material was present in the patient’s sample. These data, along with the patient’s conception history via IVF and transfer of three embryos, support the presence of chimerism.

The patient declined additional carrier screening approaches, including repeat testing using a buccal specimen or testing through an alternative laboratory. Although her partner’s carrier screening was negative, she was counseled regarding the limitations of proceeding without a maternal carrier screening result, including the inability to assess reproductive risks for X-linked conditions and the impact on linkage analysis for preimplantation genetic testing for monogenic conditions (PGT-M). The availability of diagnostic prenatal testing, including karyotype, chromosomal microarray analysis, and whole-genome sequencing, was reviewed, with discussion of the potential for test failure because of maternal cell contamination or challenges with parent-based sequencing approaches. Prenatal genetic counseling during pregnancy was recommended. The patient was also counseled on additional implications of possible chimerism, including potential challenges with organ donation or transplantation, germline genetic testing (e.g., cancer predisposition testing), and kinship analysis.

The patient pursued embryo banking and underwent controlled ovarian hyperstimulation and IVF. Six oocytes were retrieved, and five mature oocytes were exposed to sperm by conventional insemination. Three resulting blastocysts were biopsied for preimplantation genetic testing for aneuploidy (PGT-A) and two blastocysts were euploid (both 46,XX) and the third was aneuploid (47,XX +19).

## Discussion

Given the previously routine practice of multiple embryo transfer, there are likely many undiagnosed cases of chimerism. It has been theorized that the physical manipulation and close proximity of embryos throughout IVF protocols may increase the risk of amalgamation to form tetragametic chimeras (1). This complication of ART is underrecognized and therefore not a routine part of counseling with multiple embryo transfers.

Many patients may be unaware of details on how they were conceived or if their gestation involved the transfer of multiple embryos at once. Patients who were conceived via IVF before 1998 likely were one of multiple embryos that were transferred. These patients are now at the age where evaluation for infertility or other medical conditions may reveal previously unrecognized congenital chimerism.

The mechanism underlying the patient’s genetic variation is unknown. Blood group chimerism between the patient and her male twin is plausible. An additional explanation could be tetragametic fusion chimerism resulting from the fusion between the third embryo that was transferred and our patient. Tetragametic chimeras appear to be rare, with less than 100 cases reported in the literature, only two of which are noted to have been conceived through IVF (6, 7, 22). Alternatively, low-level or tissue-restricted mosaicism, defined by postzygotic chromosomal divergence within a single fertilization event, represents another possibility (1, 23). Comprehensive genotyping of the patient and her twin utilizing advanced molecular techniques such as single-nucleotide polymorphism (SNP) array analysis or short tandem repeat profiling across multiple tissues would be required to determine the exact mechanism (3, 6, 8, 9, 10, 12, 22, 24, 25).

Despite the general consideration that the clinical risk of chimerism remains low, rare cases of infertility and differences in sex development have been reported (6, 7, 10). Should a patient with chimerism require ART, additional testing or modifications may be necessary, such as a genetic evaluation from multiple tissue types, germ cell genotyping, and enhanced counseling (26, 27). Chimerism may make establishing linkage difficult or impossible, leading to inconclusive PGT-M results. If this technology is needed for pathological genetic variants, patients and physicians may consider generating a higher number of embryos or involving additional family members for linkage analysis (28). Limitations may also impact both noninvasive prenatal testing and diagnostic testing, in which maternal cell populations may increase the risk of inconclusive results or misinterpretation.

Given the potential for sex-chromosome discordance in chimerism, gonadal malignancy risk should be considered during clinical evaluation. Although gonadal malignancy risk has been characterized primarily in the disorders of sex development literature, associated risk factors include the presence of Y-chromosome sequences linked to gonadoblastoma, gonadal location, and gonadal dysgenesis (29, 30). Although gonadal malignancy risk has been reported in phenotypic females with Y-chromosome material detected in peripheral blood, these observations largely derive from cohorts referred for evaluation of symptomatic differences in sex development (31, 32). In the absence of clinical or imaging findings suggestive of gonadal dysgenesis, prophylactic gonadectomy is not routinely recommended, and management should be individualized with an emphasis on genetic counseling and clinical surveillance (29, 30).

As personalized medicine advances, genomic information is being leveraged to analyze patients’ individual risks and guide clinical decisions. Chimerism introduces unique challenges to the interpretation and integration of bioinformatic data. These challenges are exemplified by our patient’s experience utilizing 23andMe, in which she was unable to obtain information regarding her ancestry, health risks, or pharmacogenomics. Genetic counseling should convey the potential for misleading results and the need for additional testing. Several cases of chimeras have been identified in cases of disputed parentage, underscoring the potential for emotional and legal distress for families (24). This case also demonstrates gender and sex-chromosome discordance and emphasizes the need for nuanced ethical, clinical, and social approaches to discussions and management of gender and sex.

Chimerism is additionally believed to have immunologic implications. Research thus far has focused on the establishment of the role of microchimerisms in the etiology of autoimmune diseases, such as the association with parous women, and reference the pathophysiology of iatrogenic chimeras and chronic graft-vs,-host disease (33). Congenital chimerism has been shown to expand central tolerance and may present potential advantages in transplant acceptance (9). Systematic studies on the immunologic profiles and long-term clinical outcomes of patients with congenital chimerism are limited and more research is needed.

## Conclusion

We describe a case of suspected congenital XX/XY chimerism detected on routine genetic screening of a phenotypically normal, healthy female patient who was conceived via IVF with transfer of multiple embryos. This case adds to a small number of case reports describing chimeras or suspected chimerism in IVF conceptions (6, 7, 14, 15). As genetic carrier screening and direct-to-consumer genetic testing become more ubiquitous, more inconclusive results may be reported. Patients and their medical teams should be aware of the potential for chimerism as an explanation to avoid erroneous interpretations of genetic data. The implications of chimerism remain incompletely understood, particularly as advances in genomics increasingly inform medical practice. Finally, given that chimeras can result from multiple embryo transfer, we strongly advocate for the continued practice of single embryo transfer not only to minimize the risks of multifetal pregnancy, but also to avoid complications such as embryonic fusion leading to chimerism with unknown future implications.

## Declaration of Interests

A.P. has nothing to disclose. E.E. has nothing to disclose. Z.L. has nothing to disclose. E.C.F. is a consultant for Natera, Inc and Ferring Pharmaceuticals.

## References

[1] Madan K. (2020). Natural human chimeras: a review. Eur J Med Genet.

[2] Malan V., Vekemans M., Turleau C. (2006). Chimera and other fertilization errors. Clin Genet.

[3] Wenk R.E. (2018). A review of the biology and classification of human chimeras. Transfusion (Paris).

[4] Sudik R., Jakubiczka S., Nawroth F., Gilberg E., Wieacker P.F. (2001). Chimerism in a fertile woman with 46,XY karyotype and female phenotype: case report. Hum Reprod.

[5] Van De Leur S.J.C.M., Zeilmaker G.H. (1990). Double fertilization in vitro and the origin of human chimerism. Fertil Steril.

[6] Strain L., Dean J.C.S., Hamilton M.P.R., Bonthron D.T. (1998). A true hermaphrodite chimera resulting from embryo amalgamation after in vitro fertilization. N Engl J Med.

[7] Simon-Bouy B., Plachot M., Mokdad A., Lavaud N., Muti C., Bazin A. (2003). Possible human chimera detected prenatally after in vitro fertilization: a case report. Prenat Diagn.

[8] Drexler C., Wagner T. (2006). Blood group chimerism. Curr Opin Hematol.

[9] Yunis E.J., Zuniga J., Romero V., Yunis E.J. (2007). Chimerism and tetragametic chimerism in humans: implications in autoimmunity, allorecognition and tolerance. Immunol Res.

[10] Hercent A., Amar E., Valent A., Belloc S., Ferraretto X., Hermieu J.-F. (2020). Various genital and reproductive phenotypes in 46,XX/46,XY chimeras. Sex Dev.

[11] van Dijk B.A., Boomsma D.I., de Man A.J.M. (1996). Blood group chimerism in human multiple births is not rare. Am J Med Genet.

[12] Tippett P. (1983). Blood group chimeras: a review. Vox Sang.

[13] Practice Committee of the American Society for Reproductive Medicine (2012). Multiple gestation associated with infertility therapy: an American Society for Reproductive Medicine Practice Committee opinion. Fertil Steril.

[14] Williams C.A., Wallace M.R., Drury K.C., Kipersztok S., Edwards R.K., Williams R.S. (2004). Blood lymphocyte chimerism associated with IVF and monochorionic dizygous twinning: case report. Hum Reprod.

[15] Kühl-Burmeister R., Simeoni E., Weber-Matthiesen K., Milde A., Herwartz C., Neppert J. (2000). Equal distribution of congenital blood cell chimerism in dizygotic triplets after in-vitro fertilization: case report. Hum Reprod.

[16] Adamson G.D., Norman R.J. (2020). Why are multiple pregnancy rates and single embryo transfer rates so different globally, and what do we do about it?. Fertil Steril.

[17] Jain T., Missmer S.A., Hornstein M.D. (2004). Trends in embryo-transfer practice and in outcomes of the use of assisted reproductive technology in the United States. N Engl J Med.

[18] Dickey R.P. (2007). The relative contribution of assisted reproductive technologies and ovulation induction to multiple births in the United States 5 years after the Society for Assisted Reproductive Technology/American Society for Reproductive Medicine recommendation to limit the number of embryos transferred. Fertil Steril.

[19] American Society for Reproductive Medicine, Practice Committee Opinion (1998). Guidelines on number of embryos transferred.

[20] Martin J.A., Osterman M.J.K. (2024).

[21] Kulkarni A.D., Jamieson D.J., Jones H.W., Kissin D.M., Gallo M.F., Macaluso M. (2013). Fertility treatments and multiple births in the United States. N Engl J Med.

[22] Chen L., Wang L., Zeng Y., Yin D., Tang F., Xie D. (2024). A prenatal case misunderstood as specimen confusion: 46,XY/46,XY chimerism. BMC Pregnancy Childbirth.

[23] Queipo G., Zenteno J., Peña R., Nieto K., Radillo A., Dorantes L. (2002). Molecular analysis in true hermaphroditism: demonstration of low-level hidden mosaicism for Y-derived sequences in 46,XX cases. Hum Genet.

[24] Yu N., Kruskall M.S., Yunis J.J., Knoll J.H.M., Uhl L., Alosco S. (2002). Disputed maternity leading to identification of tetragametic chimerism. N Engl J Med.

[25] Shin S.Y., Yoo H.-W., Lee B.H., Kim K.S., Seo E.-J. (2012). Identification of the mechanism underlying a human chimera by SNP array analysis. Am J Med Genet A.

[26] Magharehabed M., Almadani N., Askari M., Naji M., Akbari A., Gourabi H. (2019). Rare case of an oligospermic male with 46,XX/46,XY tetragametic chimerism. Andrologia.

[27] Cheng D., Lu C.-F., Gong F., Du J., Yuan S., Luo K.-L. (2024). A case report of a normal fertile woman with 46,XX/46,XY somatic chimerism reveals a critical role for germ cells in sex determination. Hum Reprod.

[28] Practice Committee and Genetic Counseling Professional Group Of The American Society For Reproductive Medicine (2023). Indications and management of preimplantation genetic testing for monogenic conditions: a committee opinion. Fertil Steril.

[29] Hannema S.E., O’Connell M.A. (2025). Gonadectomy in individuals with a difference of sex development—for whom, when, why, and why not?. Best Pract Res Clin Endocrinol Metab.

[30] Giri D., Yewale S., Hickingbotham H., Williams C., Shalaby M., Alderson J. (2026). Mixed gonadal dysgenesis: a comprehensive review of clinical spectrum, diagnostic strategies, and management approaches. Clin Endocrinol (Oxf).

[31] Liu A.-X., Shi H.-Y., Cai Z.-J., Liu A., Zhang D., Huang H.-F. (2014). Increased risk of gonadal malignancy and prophylactic gonadectomy: a study of 102 phenotypic female patients with Y chromosome or Y-derived sequences. Hum Reprod.

[32] Huang H., Wang C., Tian Q. (2017). Gonadal tumour risk in 292 phenotypic female patients with disorders of sex development containing Y chromosome or Y-derived sequence. Clin Endocrinol (Oxf).

[33] Johnson B.N., Ehli E.A., Davies G.E., Boomsma D.I. (2020). Chimerism in health and potential implications on behavior: a systematic review. Am J Med Genet A.

